# The Relationship between Sleeping Position and Sleep Quality: A Flexible Sensor-Based Study

**DOI:** 10.3390/s22166220

**Published:** 2022-08-19

**Authors:** Yuan Zhang, Aiping Xiao, Tianhao Zheng, Huafei Xiao, Ruiyan Huang

**Affiliations:** 1 School of Technology, Beijing Forestry University, Beijing 100083, China; 2 School of Modern Equipment Manufacturing, Chenzhou Vocational Technical College, Chenzhou 423000, China

**Keywords:** flexible sensor, sleeping-position preference, turnover frequency, sleep quality

## Abstract

The use of flexible wearable sensors to monitor the impact of sleeping position and turning frequency on sleep and to study sleep patterns can help bedridden patients heal and recover. The flexible wearable sleeping-position monitoring device was designed and developed using a flexible angle sensor and a six-axis motion sensor to measure the dynamic changes in body posture during sleep. Based on the changes in the output parameters of the flexible angle sensor and the six-axis motion sensor, we determined the change in the subject’s lying position, verifying and analyzing the relationship between lying position preference, turning frequency, and sleep quality in healthy subjects. The sleeping-position monitoring device was worn by 13 subjects (7 males and 6 females) without sleep disorders before the sleep experiment. They performed more than 50 sleeping-position changes to ensure the accuracy of the monitoring device. Subjects slept in their beds for 8 h per night for 15 nights. During that time, they wore the sleeping-position monitoring device and a wristband sleep-monitoring bracelet on their left hand, and gathered the subjective sleep data using questionnaires. The results show that the most critical influencing factors are sleeping-position preference and frequency of turning. Data analysis reveals that subjects with a preference for right-sided lying and a lower frequency of turning had better sleep quality.

## 1. Introduction

Sleep’s most crucial function is recovery. Good sleep promotes healing, aids in the recovery of the immune, neurological, skeletal, and muscular systems, and is required for bedridden patients to recover [1,2]. Healthy sleep improves one’s health by allowing muscles and discs to recover from the day’s almost continuous load [3]. Sleep quantity and quality have an impact on an individual’s mood, conduct, and quality of life [4]. According to the data for 2015, more than a quarter of Chinese citizens suffer from sleep issues, and nearly a tenth suffer from sleep deprivation, with Chinese adults obtaining an average of 7.8 h of sleep per day, and the average length of sleep per day declining with age [5]. Sleep duration is associated with a variety of health problems, including cardiovascular disease, stroke, incidental arterial calcification, and changes in inflammatory markers [6]. Chronic sleep deprivation raises the risk of developing cardiovascular disease, obesity, and impaired glucose regulation, all of which impair judgment and cognition [6,7].

Today’s ergonomic bedding system design is concerned with the impact of sleep surfaces on sleep quality. The sleeping surface influences the level of sleep discomfort, and altering mattresses based on sleeping position can minimize skeletal muscle discomfort and improve sleep quality [8]. Jacobson discovered that a medium firmness mattress reduced back discomfort, low back pain, bedridden pain, and rising pain by comparing data from patients sleeping in their bed with a new bed over 28 consecutive days [9,10]. However, the mattress hardness was not quantified, and only terms such as medium firmness are used. Kovacs used European standards to assess mattress firmness, comparing a firm mattress with Hs = 2.3 to a medium-firm mattress with Hs = 5.6. Over 90 days, patients with a medium-firm mattress experienced less pain in bed and less ascending pain than those with a firm mattress [11]. Nachemson discovered that an appropriate firm mattress kept the spine in its natural unloaded state, assisting with muscle relaxation and disc rehydration [12,13,14].

Although some studies have looked at the effect of mattress firmness on spinal alignment [4,9], few studies have looked at the effect of changes in sleeping position (changes in spinal alignment) on sleep quality. Dolan discovered that when the lumbar spine flexes during postural changes, it reduces lumbar lordosis, which increases back muscle activity but reduces spinal compression and the incidence of lumbar disc disease [15]. According to Lahm and Iaizzo, mattress internal pressure caused significant changes in spinal alignment but not electromyographic changes [4]. The authors concluded that the spine was better for neutral with moderate and higher mattress internal pressure, and subjects felt more comfortable. The body contour and pressure distribution in the sleep system are highly individualized, with the body contour on the mattress surface dependent on the individual’s sleeping position. The bedding system allows body movement during the sleep cycle [16], and the body’s sleeping position changes frequently. Koninck studied sleeping-position changes in five age groups and discovered that individual body movements during sleep decreased with age, and the sleeping-position preference also gradually changed with body development [17]. DeVocht assessed the maximum pressure on the mattress in the supine position and the degree of spinal distortion in the side-sleeping position using two measures, pressure distribution and photographic comparison, and discovered that the bed pressure was directly related to the subject’s weight, with the maximum pressure being in the pelvic region [9]. However, no link has been established between spinal distortion caused by mattress deformation and sleep quality. In general, a sleep support system would provide adequate mechanical support to ensure natural spinal alignment under ideal conditions [3,9]. Deun’s use of a personalized, adaptive bedding system to optimize the subjects’ spinal alignment at night increased the length of subjects’ slow-wave sleep and improved sleep quality [18]. However, for different populations, bedding systems should be designated based on individual differences, such as obese or overweight people, who require more hardness and support [8].

Existing sleep monitoring systems mainly include polysomnography (PSG), wristband sleep monitors, and non-contact sleep-monitoring devices. Huo Yang found that the total sleep time correlation between the two was higher in younger, normal subjects by comparing the sleep parameters of wristband sleep monitors and PSG [19]. Asher used sleep-related data calculated by the EarlySense sensor, a non-contact monitoring system, to compare with the data generated by polysomnography sleep monitoring and was found to have a sleep detection accuracy of 90.5% [20]. As a result, portable, wearable sleep monitors are more convenient and require less professionalism from the user in a non-professional laboratory setting.

The above studies have concentrated on the effect of mattress biomechanical properties on sleep. While the impact on spinal alignment has been considered, there is a lack of quantitative analysis of the relationship between subject sleeping-position preferences and sleep quality. Aside from the biomechanical parameters of the mattress in a sleep system, the sleeper’s human traits can also influence sleep quality. With the rapid development of robotic bionics and the increasing demand for flexible thin-film curvature sensors, flexible wearable electronics have enormous potential for monitoring human movement and physical health [21]. The purpose of this study is to use flexible sensors and six-axis motion sensors to measure changes in body posture, body flip angle, and acceleration changes during sleep, to determine the subject’s sleeping position and the number of flips, and to verify the accuracy and reliability of the wearable measurement device. The relationship between sleep quality, sleep position preference, and frequency of turning over is explored, under the premise of ensuring the reliability of the measurement device. This method measures the dynamic changes in body posture resulting from the interaction between the sleep system and the human body, regardless of the sleeping position and the environment, and analyzes the factors affecting sleep quality in terms of changes in sleeping position.

## 2. Study of a Flexible Wearable Sleeping-Position Monitoring Device

### 2.1. Structure of the Flexible Wearable Sleeping-Position Monitoring Device

**Figure 1:**
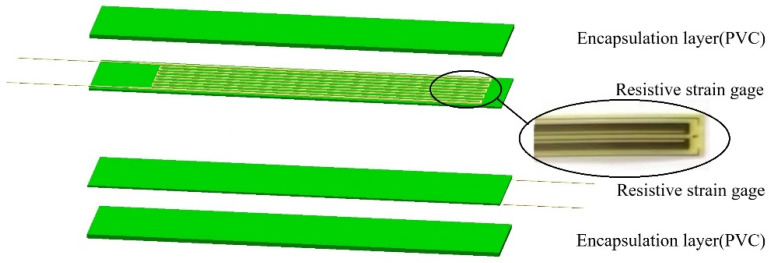
Flexible angle sensor structure diagram.

The flexible wearable sleeping-position monitoring device is designed to detect changes in human trunk posture during sleep, which uses flexible resistive strain gauges encapsulated to measure the dynamic changes in the subject’s sleeping position, with a non-adhesive, film-like curvature sensor from the Chinese Academy of Sciences as a reference [22]. The designed flexible angle sensor’s structural decomposition is shown in Figure 1: the multilayer structure is made up of two resistive flexible angle sensors (resistive strain Gages) with external leads, and two encapsulation layers located at the top and bottom of the structure. The encapsulation layer is polyvinyl chloride (PVC), the resistive flexible angle sensor sensitive grid is constantan foil, and the substrate is phenolic epoxy resin, with fine copper wires as external leads. The strain sensitivity is usually characterized by the strain coefficient (GF) given by the relative change in resistance ratio to the applied strain. For the sensor we chose a metal foil strain gauge with GF = 2, model BX120-100AA, whose sensitivity is 2.0 ± 1%, and an applicable temperature is −10 °C to 60 °C. The number of cycles at room temperature can reach 1 × 10^7^, the strain limit is 2000 με, and the insulation resistance at room temperature goes up to 10,000 MΩ, with temperature self-compensation. Metal foil strain gauges have a low cost and are technically mature, making them suitable for a wide range of applications. The flexible angle sensor adheres to the body’s surface. When the subject’s posture changes, the flexible angle sensor bends and deforms, and the conductivity of the sensitive grid material changes with the change in deformation, resulting in fluctuations in the output measurement signal.

**Figure 2:**
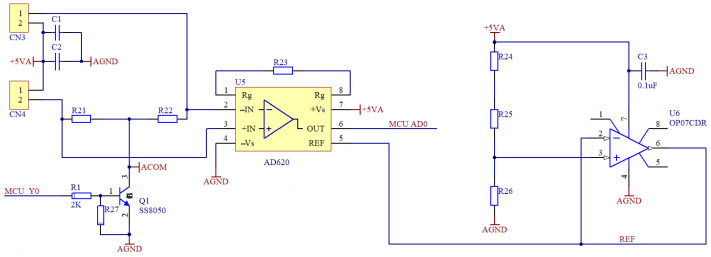
Measurement signal amplification circuit diagram.

The measurement process ignores the tensile strain of the resistive flexible angle sensor and outputs the measured signal after temperature compensation and amplification. Figure 2 depicts the measurement signal amplification circuit. The resistive flexible angle sensor is connected to the two terminals on the left end, and one of the resistive strain gauges serves as temperature compensation. The flexible angle sensor, along with the two resistors, forms a Wheatstone bridge, and the AD620 is used to amplify the output voltage, with the amount of amplification determined by resistor R23. The amplified signal’s initial output voltage ranges from −1 V to +1 V. As a result, the reference voltage of the AD620 is raised to 2 V to eliminate power supply fluctuations and reduce measurement errors. The amplified signal’s output voltage is increased to 1–3 V to ensure that the output of the amplified signal is within the microcomputer’s output range.

**Figure 3:**
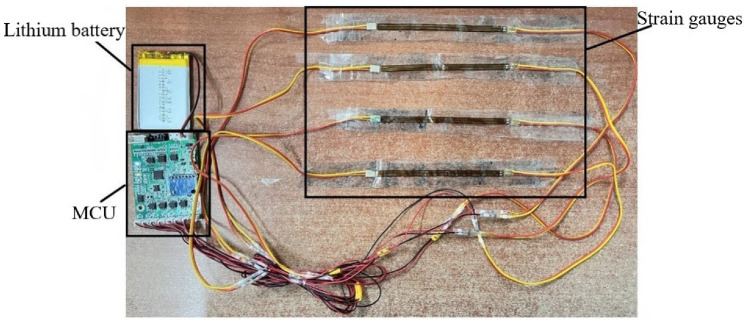
The sleeping-position monitoring device sensor, lithium battery, and MCU.

**Figure 4:**
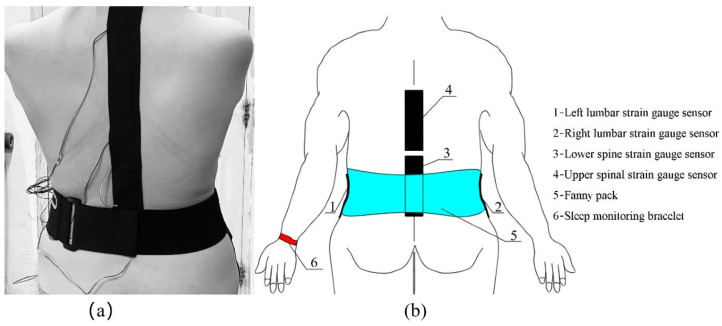
Diagram of the subject wearing the sensor: (**a**) the actual diagram of the subject wearing the sleeping-position monitoring device; (**b**) the schematic diagram of the subject wearing the sensor. When the subject’s sleeping position changes, the four flexible angle sensors bend; thus, the output voltage signal fluctuates as the sleeping position changes. The six-axis motion sensor in the waist pack changes the *x*-axis acceleration and angular velocity in response to the turning movement.

**Figure 5:**
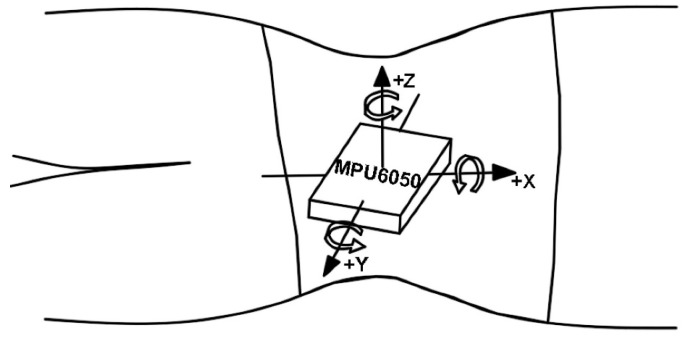
The six-axis motion sensor is oriented in the positive direction of the three coordinate axes on the subject’s body, with the *x*-axis in the direction of spinal alignment. The subject completes the turning motion with the *x*-axis as the rotation axis, the *z*-axis being perpendicular to the plane of the subject’s body, the *y*-axis being vertical to the *x*-axis, and the right-hand rule being satisfied.

Figure 3 shows the sensor, lithium battery, and micro control unit (MCU) of the sleeping-position monitoring device, with the strain gauge sensor attached to the subject’s left and right lateral lumbar, as well as to the upper and lower spine, by a muscle patch, and the MPU6050 six-axis motion sensor for monitoring the turning movement is located on the MCU module. The sensors are controlled and communicated with via the MCU module to collect and store the information about the subject’s sleeping position change. The diagram of the subject wearing the sleeping-position monitoring device is shown in Figure 4. The six-axis motion sensor MPU6050, which monitors turning movement, is strapped to the belly button via a waist pack. As shown in Figure 5, the six-axis motion sensor is oriented in the three axes of the subject’s body, with the *X*-axis of the motion sensor serving as the axis of rotation when the subject turns over. The *X*-axis acceleration and angular velocity fluctuate considerably when turning over. Because of the change in sleeping position, the flexible angle sensors attached to the subject’s upper and lower spine, the left and right lateral lumbar regions, bent, and the measurement circuit’s output voltage fluctuated significantly.

**Figure 6:**
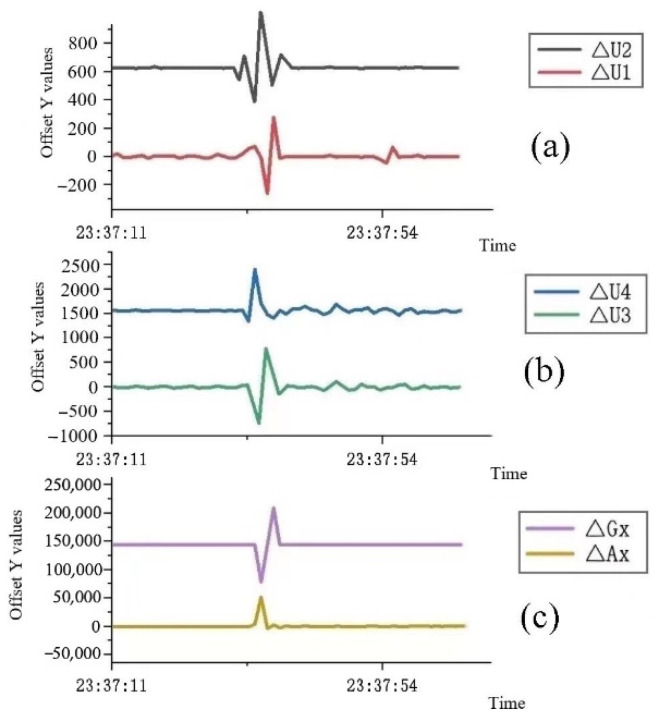
Analog output fluctuation graph of each sensor during the turning-over action: (**a**) the analog output voltage change curves of the left and right lateral lumbar flexible angle sensors, ∆U1 and ∆U2, are the analog output voltage change values of the left and right lumbar flexible angle sensors after bending, respectively; (**b**) the analog output voltage change curves of the upper and lower spine flexible angle sensors, where ∆U3 and ∆U4 are the analog output voltage change values of lower and upper spine flexible angle sensors after bending, respectively; (**c**) the acceleration and angular velocity analog output change curves of the six-axis motion sensor, where ∆Ax and ∆Gx are the lumbar motion sensor’s analog output acceleration and angular velocity change values, respectively.

The large fluctuations in the analog output waveforms of the flexible angle and six-axis motion sensors at the same time, as shown in Figure 6, can be used to determine that the subject has rolled over and that the subject’s sleeping position has changed by referring to each subject’s 12 types of sleeping position change data. Each subject was subjected to 12 sleeping-position changes in turn. For each sleeping position, the positive and negative values of ∆U1 and ∆U2 for the left and right side of the waist, as well as ∆U3 and ∆U4 for the lower and upper spinal strain gauges, were different due to the different directions of the upper and lower spine and left- and right-side waist bending. For example, when the subject is lying on his or her left side, the left lumbar flexure sensor will sag and bend, the right lumbar flexure sensor will be close to horizontal, while the upper and lower spine flexure sensors have a certain degree of bending due to the individual’s arched back; when the subject is lying on his back, the upper and lower spine flexure sensors are close to horizontal, while the left and right lumbar flexure sensors have a certain bending deformation. Due to each subject’s differences, the data of the 12 sleeping-position changes were collected from each subject. The number of sleeping-position changes and the sleeping position of the subject during the whole night could be judged according to their sleeping-position change data. Since the subject’s body was twisted and repeatedly moved during the turning process, the signal would fluctuate, so there would be several peaks when there was a turning action. Nonetheless, the signal would not fluctuate for some time after the turning action was completed, and the maximum peak was used to judge the subject’s lying position change.

### 2.2. Experimental Procedure for the Reliability of the Flexible Wearable Sleeping-Position Monitoring Device

A total of 13 subjects (7 males and 6 females; mean age 24 ± 2 years) were included; each subject was required to attach a flexible angle sensor and use their sheets and pillows while using the same style of bed and mattress to reduce the influence of environmental factors. Before the experiment, each subject was attached to the flexible angle sensor at the left and right lateral waist as well as the upper and lower spine, respectively, and the main control circuit board and motion sensor were strapped to the waist with a waist pack, and the six-axis motion sensor was secured at the navel position. After the subject was laid down on the bed, the position of the flexible angle sensor was finely adjusted to ensure the comfort of the subject and the accuracy of the measurement results.

Each subject changed their sleeping position in sequence according to the following 12 postural variations, and once completed, they could change their posture at will: supine to prone, prone to left lateral, left lateral to supine, supine to right lateral, right lateral to left lateral, left lateral to prone, prone to right lateral, right lateral to supine, supine to left lateral, left lateral to right lateral, left lateral to right lateral, right lateral to prone, and prone to supine.

According to the data of the 12 posture changes of each subject, the values of ∆U1, ∆U2, ∆U3, and ∆U4, corresponding to the flexible angle sensors of the left and right lateral lumbar and lower and upper spine of the subject, were different when the subject was in different sleeping positions, and the values of the *x*-axis acceleration ∆Ax and angular velocity ∆Gx of the six-axis motion sensors were also different for different sleeping-position changes. To ensure adequate data, each subject was required to change sleeping positions more than 50 times during the reliability experiments. To verify the accuracy of the flexible wearable sleeping-position monitoring device, 13 subjects were monitored using a video device throughout the sleeping-position changes. The six parameters measured by the flexible wearable sleeping-position monitoring device were compared with its own 12 sleeping-position changes to determine which sleeping-position change the subject was in, and the results were compared with the subject’s sleeping-position changes in the video monitoring to verify the accuracy and reliability of the flexible wearable sleeping-position monitoring device. Everyone may have slight differences due to the specific position in which the sleep monitoring device is bound, so when conducting individual sleep-data collection experiments, it was necessary to record the sensor data for these 12 changes in the sleep position to facilitate the later identification of which sleeping position the subject was in.

### 2.3. Statistical Analysis of the Reliability of the Flexible Wearable Sleeping-Position Monitoring Device

The device’s accuracy was determined by the correct judgment rate of the turning-over movements and the number of turning-over movements. If the accuracy rate exceeds 90%, the device was proven to be highly accurate and reliable. Furthermore, a significance test result of “*p* = 0.05” indicated statistical significance. Unless otherwise specified, results are expressed as the mean ± standard deviation.

### 2.4. Validation of the Reliability Results of the Flexible Wearable Sleeping-Position Monitoring Device

**Figure 7:**
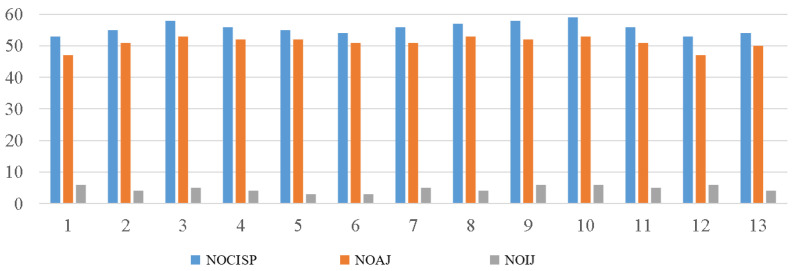
Reliability diagram for the flexible wearable sleeping-position monitoring device. Note: NOCISP = number of changes in sleeping position; NOAJ = number of accurate judgments; NOIJ = number of incorrect judgments.

The data and the number of correct judgments of changes in sleeping position of 13 subjects wearing the sleeping-position monitoring device are shown in Figure 7. The *t*-test analysis of the experimental data on the reliability of the flexible wearable sleeping-position monitoring device for the 13 subjects revealed a statistically significant correlation between the number of sleeping-position changes and the number of correct judgments (r = 0.95, *p* < 0.001). The subject had more than 50 sleeping postural changes. Based on the data sheets of each sensor for each of the 12 sleeping postural changes of each subject, the type of sleeping postural change of each subject was determined, and the results were compared to the subjects’ video-monitored sleeping postural changes to confirm the accuracy of the postural change measurements and to determine the device’s accuracy. The average number of sleeping postural changes was 55.69 ± 1.93, the number of correct judgments was 51.00 ± 2.00, and the number of incorrect judgments is 4.69 ± 1.11; thus, the accuracy rate was 91.58% ± 1.98%. The data analysis shows that the accuracy of the flexible wearable sleeping-position monitoring device is high, exceeding 90%; therefore, it can be used to collect data for sleep experiments.

## 3. Experimental Study on the Effect of Sleeping-Position Change on Sleep

### 3.1. Sleep Effects of Changes in Sleeping Position

Before the experiment, 13 subjects who had participated in the reliability validation of the flexible wearable sleeping-position monitoring device were required to read the sleep-experiment protocol and complete the Pittsburgh Sleep Quality Index form [23]. All subjects had a habit of wearing a sleep-monitoring bracelet and were in good health, with normal sleep, no insomnia, and no behaviors that might interfere with normal sleep. Examples include taking sleep medication, anti-depressants, caffeine-based drinks, foods, and any form of back pain. In addition, each subject signed an informed consent form.

### 3.2. Experimental Design for Sleep Effects of Changes in Sleeping Position

For the sleep experiment, each participant wore a flexible wearable sleeping-position monitoring device. They completed the sleep experiment under the premise of verifying the reliability of the measuring device.

### 3.3. Experimental Procedure for the Effect of Changes in Sleeping Position on Sleep

The sleep measurement experiment was carried out after the device’s reliability and accuracy was confirmed. On each reference night, each subject was subjected to the same experimental conditions. Subjects had to wear the sleep-monitoring device shown in Figure 2 and the sleep-quality-monitoring bracelet on their left hand. Subjects slept in their bedrooms, using their sheets and pillows but the same style of bed and mattress, and sleeping in their preferred position in bed, to reduce the influence of external environmental factors on sleep quality.

Subjects slept for 7.5 to 9 h per night, with a bedtime between 23:00 and 24:00, and were awakened between 7:30 and 8:30 a.m. Subjects were required to power up and power down the device before going to bed and after waking up and to record the time of power up and power down. Subjects must not drink caffeinated beverages, exercise vigorously, or overeat in a way that interferes with sleep during the three hours before bedtime, but they may engage in appropriate recreational activities such as walking, watching television, reading, and talking. During sleep, subjects wore a sleep-monitoring bracelet and a sleeping-position monitoring device to record their objective sleep parameters. To assess subjective sleep quality and mood changes, subjects also completed the Karolinska Sleepiness Scale (KSS) and the Mood Scale (MS) at 23:15 before bedtime and 8:45 after awakening in the morning of each reference night.

### 3.4. Objective and Subjective Sleep-Quality Measures

Because polysomnography has a strong first-night effect, subjects have difficulty falling asleep when using such devices for the first time, affecting the analysis and judgment of sleep parameters. There was no significant first-night effect for monitoring sleep quality with the wristband sleep-monitoring bracelets, and there were no significant differences in any baseline nighttime sleep variables [24]. To reduce the impact of other factors, all subjects wore a sleep-monitoring bracelet daily. Sleep-monitoring bracelets were used for sleep monitoring due to issues such as the difficulty of carrying such devices. Even though wristband sleep-monitoring bracelets have not yet entered clinical medicine, sleep monitoring in everyday life has met the demand with high overall correctness and sensitivity [25]. Subjects were asked to complete 15 full nightly sleep measurements, and a sleep-monitoring bracelet was worn on the subject’s left wrist to track objective sleep quality. The wristband sleep-monitoring bracelet analyzes the level of physical activity based on the wearer’s wrist activity and heart rate to determine the state of sleep the wearer is in, with a sampling frequency set at 60 Hz.

In addition to the objective measurement of sleep, subjects were asked to complete a Pittsburgh Sleep Quality Index form before participating in the sleep experiment to determine the absence of sleep disorders. The Pittsburgh Sleep Quality Index (PSQI) is the only clinically available broad index that covers a wide range of sleep-quality indicators and has strong reliability and validity in sleep-quality analysis to achieve the desired results [26]. Subjects were asked to complete the Karolinska Sleepiness Scale and the Mood Scale before bedtime and after waking up to assess subjective sleep quality and morning and evening mood swings. To begin, the KSS was used to assess subjective sleepiness. The difference in sleepiness between morning and evening allowed for the evaluation of sleep quality and whether it relieved the subject’s sleepiness [27]. Second, the MS was used to reflect differences in negative moods between the morning and evening. Given that both scales were used to assess negative mood, the positive difference between these two scales’ evening and morning scores indicated a night of excellent sleep quality. The more significant the positive difference and the better the subjective sleep quality, the lower the morning score compared to the evening score.

### 3.5. Statistical Analysis of Experimental Data on the Effect of Changes in Sleeping Position on Sleep

First, the objective sleep data were normally distributed. The mean was determined by excluding the effects of other variables using a parametric repeated-measures ANOVA. Second, due to the ordinal nature of the subjective scale, the personal sleep data were non-normally distributed and were analyzed using a non-parametric Friedman ANOVA. Furthermore, a *p*-value < 0.05 indicated statistical significance. Unless otherwise specified, results are expressed as the mean ± standard deviation.

### 3.6. Experimental Results on the Effect of Changes in Sleeping Position on Sleep

**Figure 8:**
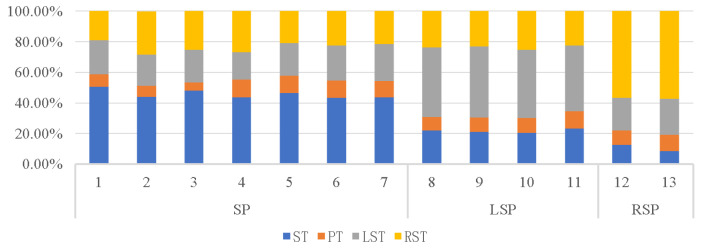
Percentage of sleep time in subjects with different sleeping-position preferences.

**Figure 9:**
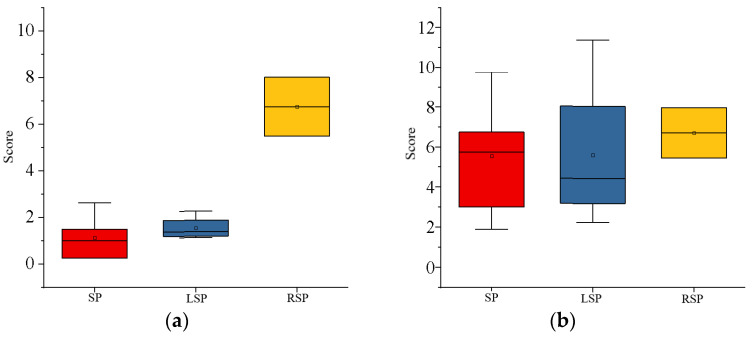
Subjective sleep-quality graph of subjects with three different sleeping-position preferences: (**a**) the difference in morning and evening sleepiness (KSS score); (**b**) the fatigue state (MS score).

The four main types of sleeping positions are supine, prone, left lateral, and right lateral. The sleeping positions preferred by the 13 subjects who took part in the study were prone (54%), left lateral (31%), and right lateral (15%), with no preference for prone. Table 1 shows the average time spent in each of the four sleeping positions by subjects with varying sleeping-position preferences. The position in which the subject spent more than 40% of the night asleep was designated as their preferred position. Figure 6 depicts the percentage of time spent in each of the four positions by the 13 subjects during the baseline night. Subjects consumed most of their sleep time in their preferred position throughout the night, as shown in Table 1 and Figure 8, while all subjects spent very little time in the prone position. Figure 9 shows that subjects with a right-sided preference had the best subjective sleep quality, subjects with a left-sided preference had the second best, and subjects with a supine preference had the worst.

**Figure 10:**
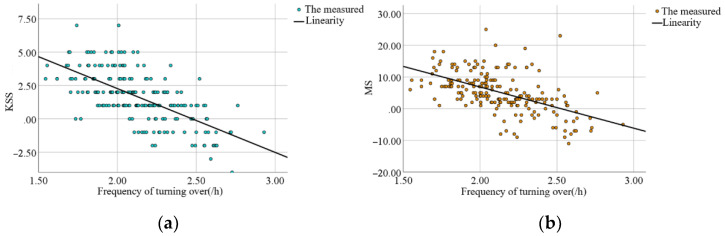
The plot of the relationship between turning-over frequency and subjective sleep quality: (**a**) the scatter plot of the difference between tossing frequency and morning and evening sleepiness (KSS score); (**b**) the scatter plot of tossing frequency and fatigue status (MS score).

On each base night, each subject had a certain amount of turning movement while sleeping. Individual factors influenced the frequency of turning over, which varied with sleep quality. The frequency of turning was highly correlated and statistically significant, with differences in the KSS scores (r = −0.65, *p* < 0.001) and MS scores (r = −0.55, *p* < 0.001). When the data for turning frequency, KSS score difference, and MS score difference were fitted to each of the 13 subjects, a relationship between subjective sleep quality and turning frequency was discovered, as shown in Figure 10. Within a certain range, the subjects’ subjective sleep quality tended to decrease as the frequency of turning over increased.

Table 2 shows the objective sleep data for subjects with different sleeping-position preferences. The time spent in bed and total sleep time were similar for the three sleeping-position preferences. In comparison, subjects with a right-sided preference experienced the fewest awakenings, followed by those with left-sided preference. Those with a supine preference experienced slightly fewer awakenings than those with left-sided preference. Subjects with a right lateral sleeping-position preference had the longest duration of slow-wave sleep, followed by the supine preference; the left lateral sleeping-position preference had the shortest period of slow-wave sleep. The duration of rapid eye movement (REM) and slow-wave sleep was opposite for the three sleeping-position preferences, with the right-sided preference having significantly less REM than the other subjects.

## 4. Discussion

The purpose of this study was to develop a flexible wearable sleeping-position monitoring device that could be used to investigate the impact of sleeping-position change on sleep quality. A sleep experiment was then used to determine the relationship between sleeping-position preference, turning frequency, and sleep quality. The findings reveal that the subjective sleep quality from high to low was right-sided, left-sided, and supine; additionally, the sleep quality of subjects without sleep disorders was inversely related to the frequency of tossing and turning, with the better the sleep quality, the lower the frequency of turning.

- The average number of posture changes of the 13 subjects in the reliability experiment of the flexible wearable sleeping-position monitoring device was 55.69 ± 1.93, the number of correct judgments was 51.00 ± 2.00, and the number of incorrect judgments was 4.69 ± 1.11; thus, the accuracy rate was 91.58% ± 1.98%. During the sleep experiment, the experiment validated the accuracy and reliability of the data measured by the flexible wearable sleeping-position monitoring device.
- Analysis of data on sleeping-position preferences showed that the supine position was preferred by more than half of the subjects, followed by the left lateral position and finally the right lateral position, with a lack of subjects in the prone position due to the small number of subjects. According to the proportion of time spent in each of the four positions, the biggest difference between subjects who prefer the three different decumbent positions was that they spent about half of their night in their preferred position and about 10% of their time in the prone position.
- Analysis of the subjective sleep-quality data revealed that sleep-position preference influenced sleep quality, with subjects in the right lateral position having the best sleep quality and those in the left lateral position having slightly higher sleep quality than those in the supine position. Subjective sleep quality was reflected in the results of the Karolinska Sleepiness Scale and the Mood Scale. Each day of the experiment, these scales were completed the night before and the morning after. Subjects who preferred to sleep on their right side scored significantly lower in the early morning of the second day than they did the night before, implying that subjects who spent most of their time sleeping on their right side recovered better after a night of rest and adjustment.
- The effect of turning frequency on subjective sleep quality showed that subjects would turn over more frequently when their sleep quality was poor. When subjects were deeply asleep, they would turn less frequently, and when they first went to bed, they would turn over frequently to find a suitable position to fall asleep, but the preferred position was not always the one in which they fell asleep. Individual variation in turning frequency precludes fitting a mathematical equation. The frequency of turning in subjects ranged from 1.86/h to 2.40/h, and subjects could turn over dozens of times in a single night’s sleep, indicating that each individual does not stay in the same position for long periods during sleep.
- Analysis of the objective sleep data revealed that sleeping-position preference and frequency of turning interacted with sleep parameters such as multiple awakenings and the rapid eye movement period. For starters, subjects with a right-sided preference had fewer awakenings and shorter awakening times than those with other sleeping-position preferences. On the other hand, subjects with a supine preference had a slightly higher number of awakenings than subjects with a left lateral preference, with the lower awakening time being a higher number of awakenings but a shorter duration of the awakenings. In subjects who preferred to sleep supine, the increased number of awakenings may have resulted in a lower subjective sleep-quality rating. Second, subjects who preferred the right lateral position had the longest duration of slow-wave sleep and a significantly shorter duration of rapid eye movement, indicating a lower proclivity to wake up during the night.

## 5. Limitations

The limitation of this paper is that subjects may experience first-night effects, which may cause discomfort due to wearing the flexible sensor. The first night of sleep monitoring may result in poor sleep, increased anxiety, and awareness of being monitored. However, after subjects have adapted to the measurement device, the device no longer impairs sleep, and the state of stress and fatigue disappears. To avoid the impact of the first night’s data on the sleep data, the experimental data were collected by discarding the data collected on the first night.

Second, since there were subjects lacking the prone-position preference during the experiment, we were unable to determine the characteristics of their sleep quality compared to those who sleep in the prone position for extended periods. The effect of different sleeping positions and frequency of turning on sleep quality could be better analyzed if the number of subjects in subsequent experiments could be increased to cover more subjects with different sleep characteristics. Furthermore, this is the first time the flexible wearable sleeping-position monitoring device has been used in a sleep experiment, so some errors and chance findings are likely. In the future, optimizing the structure of the measuring device and further improving its professional use can be considered. Without taking individual differences into account, the effect of the bedding system on sleep was ignored by comparing the three sleeping-position preferences of subjects without sleep disorders, as well as the relationship between the frequency of turning over and subjective sleep quality of all subjects at each baseline night. Sleep quality is also affected by personal and objective factors, such as the environment, which is a complex system.

In conclusion, the findings of this study suggest that subjects without sleep disorders who prefer to sleep on their side will sleep better than those who like to sleep on their back and that a higher frequency of turning during sleep will reduce sleep quality. The difference in sleep quality between the three different sleeping-position preferences may be attributed to differences in the sleeping position in which the subject spends the majority of the night. The frequency of turning over in healthy subjects may also influence sleep quality, with frequent turning representing an uncomfortable sleeping position and subjects being more likely to awaken from sleep, thus increasing the number of awakenings. Future research could look at subjects’ different sleeping preferences and frequency of turning throughout the night as independent factors in studying sleep quality.

## Figures and Tables

**Table 1:** Percentage of sleep time in subjects with different sleeping-position preferences.

SPP	ST (%)	PT (%)	LST (%)	RST (%)
SP	45.68 ± 1.04	9.35 ± 0.93	21.43 ± 0.76	23.54 ± 1.32
LSP	21.62 ± 0.63	9.86 ± 0.52	44.86 ± 0.71	23.67 ± 0.56
RSP	10.53 ± 2.00	10.03 ± 0.59	22.35 ± 1.06	57.08 ± 0.35

Note: SPP = sleeping-position preference; SP = supine preference; LSP = left-side sleeping-position preference; RSP = right-side sleeping-position preference; TIB = time in bed; ST = supine time (%TIB); PT = prone time (%TIB); LST = left-side time (%TIB); RST = right-side time (%TIB).

**Table 2:** Subjects’ objective sleep data.

SPP	SP	LSP	RSP
TIB (min)	471.50 ± 4.94	472.15 ± 2.96	471.77 ± 7.02
SOL (min)	20.81 ± 5.05	19.47 ± 4.63	26.00 ± 0.94
TST (min)	445.20 ± 6.63	442.30 ± 6.68	441.23 ± 7.68
TWT (min)	3.69 ± 0.50	7.03 ± 3.43	4.20 ± 0.38
Awakings (n)	0.80 ± 0.46	0.75 ± 0.70	0.23 ± 0.05
Awake (min)	1.81 ± 2.11	3.35 ± 2.37	0.33 ± 0.09
SWS (min)	245.87 ± 27.93	231.40 ± 47.07	274.07 ± 5.37
REM (min)	82.59 ± 17.07	89.55 ± 3.61	70.37 ± 16.36
Awake (%TIB)	0.41 ± 0.48	0.75 ± 0.53	0.08 ± 0.02
SWS (%TIB)	55.25 ± 6.70	52.27 ± 10.24	62.15 ± 2.39
REM (%TIB)	18.52 ± 3.66	20.26 ± 1.10	15.92 ± 3.43
SEI (%)	94.43 ± 1.07	93.68% ± 1.34	93.53% ± 0.24

Note: TIB = time in bed; SOL = sleep-onset latency; TST = total sleep time; TWT = total wake-up time; SWS = slow-wave sleep time; REM = rapid eye movement; SEI = sleep-efficiency index (TST/TIB).

## Data Availability

The data covered in the text are represented in the text in the form of graphical icons.
